# Linking Cytoscape and the corynebacterial reference database CoryneRegNet

**DOI:** 10.1186/1471-2164-9-184

**Published:** 2008-04-21

**Authors:** Jan Baumbach, Leonard Apeltsin

**Affiliations:** 1 Center for Biotechnology, Bielefeld University, Bielefeld, Germany; 2 Department of Pharmaceutical Chemistry, University of California, San Francisco, San Francisco, CA, USA

## Abstract

**Background:**

Recently, the research community has seen an influx of data relating to transcriptional regulatory interactions of Corynebacteria, organisms that are highly relevant to fields of systems biology, biotechnology, and human medicine. Information derived from DNA microarray experiments, computational predictions, and literature has opened the way for the graph-based analysis, visualization, and reconstruction of transcriptional regulatory networks across entire organisms. The reference database for corynebacterial gene regulatory networks CoryneRegNet provides methods for data storage and data exchange in a well-structured manner. Additional information on the model organism *Escherichia coli *K12 obtained from RegulonDB has been integrated. Generally, gene regulatory networks can be visualized as graphs by drawing directed edges between nodes, where a node represents a gene and an edge corresponds to a typed regulatory interaction. Cytoscape is an open-source software project whose aim is to provide graph-based visualization and analysis for biological networks. Its architecture allows the development and integration of user-made plugins to enhance core functionalities.

**Results:**

We introduce two novel plugins for the Cytoscape environment designed to enhance *in silico *studies of procaryotic transcriptional regulatory networks. Our plugins leverage the information from the cornyebacterial reference database CoryneRegNet with the graph analysis capabilities of Cytoscape. *CoryneRegNetLoader *queries the CoryneRegNet database to extract a gene regulatory network represented as a directed graph. Additional information is stored as node/edge attributes within the graph. *COMA *facilitates consistency checks for gene expression studies given a gene regulatory network. *COMA *tests whether all gene expression levels correlate properly with the given network topology.

**Conclusion:**

The plugins facilitate *in silico *studies of procaryotic transcriptional gene regulation, particularly in Corynebacteria and *E. coli*, by combining the knowledge from the corynebacterial reference database with the graph analysis capabilities of Cytoscape, which is one of the most-widely used tools for biological network analyses.

## Background

Recently, the research community has seen an influx of data relating to transcriptional regulatory interactions of microorganisms. Information derived from DNA microarray hybridization experiments, computational predictions, and literature-based text mining has opened the way for the graph-based analysis of gene regulatory networks across entire organisms [1,2]. The large-scale reconstruction of these networks allows for *in silico *simulation of cell behavior in response to changing environmental conditions [3].

Corynebacteria are highly relevant to fields of systems biology, biotechnology, and human medicine [4]. The ontology-based reference database for corynebacterial transcription factors and regulatory networks CoryneRegNet [5-8] provides methods for the storage, analysis, and reconstruction of gene regulatory networks of corynebacteria. Data on the model organism *Escherichia coli *K12 has been imported from RegulonDB release 5.0 [9,10] and integrated together with corynebacterial data (refer to [8] for a detailed description of the integrated data). The reconstructed gene regulatory networks can be visualized as graphs by drawing typed, directed edges between nodes, where a node represents a gene and an edge corresponds to a known gene regulatory interaction (activation or repression).

Cytoscape [11-13] is an open-source software project whose aim is to provide graph-based visualization and analysis for all biological networks. Its architecture allows the development and integration of user-made plugins to enhance core functionalities. The easy to use plugin interface has encouraged the community to develop a number of Cytoscape plugins, which are publicly available at [14].

In this short article, we introduce two novel plugins for the Cytoscape environment. The plugins facilitate *in silico *studies of procaryotic transcriptional gene regulation, particularly in Corynebacteria and *E. coli*, by combining the knowledge from the corynebacterial reference database with the graph analysis capabilities of Cytoscape, which is one of the most-widely used tools for biological network analyses.

## Implementation

Both plugins are implemented in Java 5 in compliance with the Cytoscape plugin interface mechanism by extending the class *CytoscapePlugin *and creating Java JAR files. A tutorial on Cytoscape plugins may be found at [15]. The plugins are publicly available at the Cytoscape plugin site at [14] (*COMA *in section 'Analysis' and *CoryneRegNetLoader *in section 'Network and Attribute I/O') as JAR files, which have to be copied into the Cytoscape plugins directory. They can also be installed more easily by using the 'Plugins'/'Manage plugins' menu of Cytoscape. In order to run *CoryneRegNetLoader *internet access is necessary. The plugins are compatible with Cytoscape version 2.5.x and have also been tested with the current beta release 2.6. The URL for a documentation and a step-by-step description of an application example is given in Availability and Requirements. The source code of both plugins is included in the JAR files.

## Results and Conclusion

### CoryneRegNet plugin

The *CoryneRegNetLoader *is able to extract transcriptional regulatory networks stored in CoryneRegNet and to import them into Cytoscape. A directed graph is then created, with genes as nodes and regulatory interactions as directed edges. For each node, *CoryneRegNetLoader *imports the up-to-date gene and protein annotation data, which is stored as node attributes. For each edge, the plugin retrieves the evidence of the underlying protein-DNA interaction and whether the target genes are up- or downregulated. This data is stored as edge attributes. In [16] we described the SOAP-based Web Service server of CoryneRegNet and presented an application example that shows how the community profits from an integrated visualization of corynebacterial network data. With this plugin, we take a step forward and provide an interface to a more powerful environment for integrated biological network analyses.

### COMA plugin

The *COMA *plugin implements a method to facilitate consistency checks for gene expression studies given a gene regulatory network. It checks whether the expression levels of all genes are as expected from the network topology.

We discretize the expression levels of genes to overexpressed ('+') and underexpressed ('-') respectively. Furthermore, we do the same for the activation or repression of a target gene by a regulator. Let *g *∈ {+, -} be the gene expression of a gene *G*. Let *t *∈ {+, -} be the gene expression of a transcription factor *T*, which regulates *G*. Finally, let *r *∈ {+, -} be the known gene regulatory interaction of gene *G *by transcription factor *T*. Subsequently consider the algebraic signs in the following equation:

*t*·*g *= *r*

If the equation is incorrect, for instance '+' · '-' = '+', this is a putative inconsistency with the given regulatory network; also refer to [5]. Based on this, for every gene *G *of a given microarray experiment with expression *g*, all transcription factors *T *that regulate *G *are considered, and for all transcription factors the expression level *t *and the type of the regulatory interaction *r *is determined. Subsequently, *COMA *applies the above explained inconsistency test. Table 1 summarizes the results for all possible combinations of *t*, *g*, and *r*.

**Table 1:** Potential inconsistencies for gene regulatory interactions given a gene expression study.

TF expr. *t*	Target gene expr. *g*	Regulation type *r*	Contradiction?
+	+	-	yes
+	-	+	yes
-	+	+	yes
-	-	-	yes
+	+	+	no
+	-	-	no
-	+	-	no
-	-	+	no

This table illustrates all gene expression/regulation combinations for a transcription factor *T *(with relative expression level *t*), a target gene *G *(with relative expression level *g*), and a regulatory interaction of type *r*. For the first four rows, *COMA *would suggest that the gene regulation type *r *contradicts the given gene expression levels for the transcription factor *T *and the target gene *G*.

**Figure 1:**
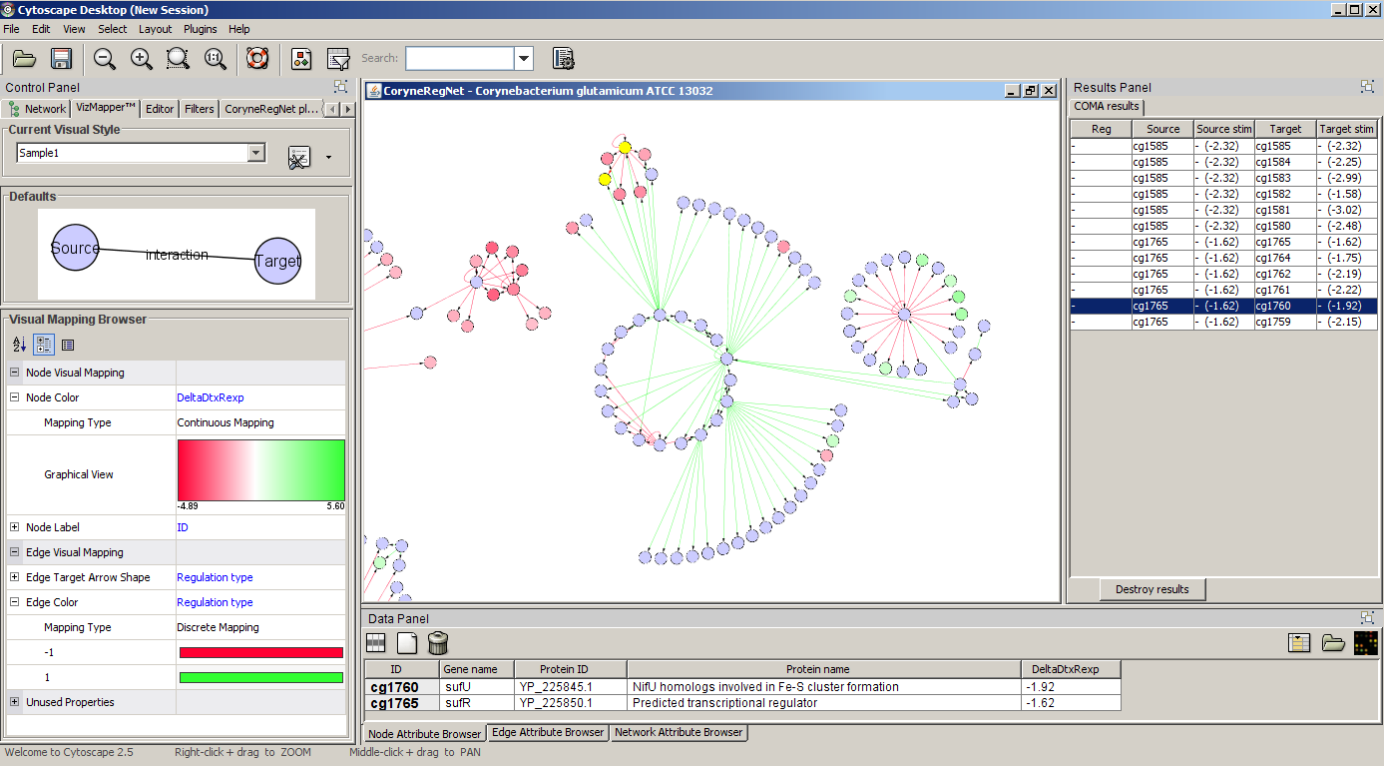
**The complete gene regulatory network of C. glutamicum visualized with Cytoscape**. This figure shows parts of the complete gene regulatory network of *Corynebacterium glutamicum *imported by using the *CoryneRegNetLoader *plugin. The two highlighted nodes in yellow are those selected from the *COMA *results table (right). Nodes correspond to genes, edges to gene regulatory interactions. Cytoscape has been configured to visualize activations as green edges and repressions as red edges respectively. The relative gene expression levels are continously mapped to the node colors: red – underexpressed, green -overexpressed, and blue – no significant differential gene expression.

Although *COMA *was designed to work with the *CoryneRegNetLoader*, it can be used with any directed biological network as long as there is a Cytoscape edge attribute of type boolean, integer, or float. This attribute is used to distinguish between up- and downregulations and can be defined by the user. Gene expression values can be imported by using the Cytoscape microarray import feature, for instance. Generally, any node attribute of type integer or float can be used for *COMA*. Each putative inconsistent edge is listed in a result table. If the user selects a certain node-pair, both nodes including the edge between them are highlighted in the Cytoscape graph. Figure 1 show a subset of the complete gene regulatory network of *C. glutamicum*, extracted from CoryneRegNet by utilizing the *CoryneRegNetLoader *plugin. The *COMA *plugin has been applied to the graph by using the results of a gene expression study provided by Brune et. al [17], which is also available in Cytoscape format as Additional file 1. With this plugin, Cytoscape now provides hints for missing regulatory interactions.

Both plugins together facilitate *in silico *the study and visualization of procaryotic transcriptional gene regulation, particularly in Corynebacteria and *E. coli*, by combining the manually curated knowledge from the CoryneRegNet database with the power of the graph analysis and visualization capabilities of Cytoscape.

Since various chemical mechanisms take part in the regulation of the genes, in a future version of the *COMA *plugin we plan to consider also non-transcriptional gene regulatory interactions. Additionally, we plan to extend *COMA *to check for coherent and incoherent feed-forward loops [18] in a given network. We will also conside further rules to validate microarray data in the context of known regulatory networks (see e.g. [19]).

## Availability and Requirements

Project name: CoryneRegNetLoader and COMA

Project home page:  and 

Documentation and application example: 

Operating system(s): Platform independent

Programming language: Java 5

License: Academic Free License (AFL)

Any restrictions to use by non-academics: User should contact Jan.Baumbach@CeBiTec.Uni-Bielefeld.DE.

## Authors' contributions

JB and LA developed and implemented the plugins. Both authors contributed to writing and both read and approved the final manuscript.

## Supplementary Material

Additional file 1 Microarray result file in Cytoscape format. The results of a gene expression study provided by [17] in Cytoscape format. Listed are the m-values for those genes that have been differentially expressed significantly. Click here for file
